# Manipulating graded exercise test variables affects the validity of the lactate threshold and ${\dot{V}O}_{2\mathrm{peak}}$

**DOI:** 10.1371/journal.pone.0199794

**Published:** 2018-07-30

**Authors:** Nicholas A. Jamnick, Javier Botella, David B. Pyne, David J. Bishop

**Affiliations:** 1 Institute for Health and Sport, College of Sport and Exercise Science, Victoria University, Melbourne, Australia; 2 Australian Institute of Sport, Canberra, Australia; 3 Research Institute for Sport and Exercise (UCRISE), University of Canberra, Canberra, Australia; 4 School of Medical and Health Sciences, Edith Cowan University, Joondalup, Australia; Norwegian University of Science and Technology, NORWAY

## Abstract

**Background:**

To determine the validity of the lactate threshold (LT) and maximal oxygen uptake (${\dot{V}O}_{2\max}$) determined during graded exercise test (GXT) of different durations and using different LT calculations. Trained male cyclists (n = 17) completed five GXTs of varying stage length (1, 3, 4, 7 and 10 min) to establish the LT, and a series of 30-min constant power bouts to establish the maximal lactate steady state (MLSS). ${\dot{V}O}_{2}$ was assessed during each GXT and a subsequent verification exhaustive bout (VEB), and 14 different LTs were calculated from four of the GXTs (3, 4, 7 and 10 min)—yielding a total 56 LTs. Agreement was assessed between the highest ${\dot{V}O}_{2}$ measured during each GXT (${\dot{V}O}_{2\mathrm{peak}}$) as well as between each LT and MLSS. ${\dot{V}O}_{2\mathrm{peak}}$ and LT data were analysed using mean difference (MD) and intraclass correlation (ICC).

**Results:**

The ${\dot{V}O}_{2\mathrm{peak}}$ value from GXT_1_ was 61.0 ± 5.3 mL^.^kg^-1.^min^-1^ and the peak power 420 ± 55 W (mean ± SD). The power at the MLSS was 264 ± 39 W. ${\dot{V}O}_{2\mathrm{peak}}$ from GXT_3, 4, 7, 10_ underestimated ${\dot{V}O}_{2\mathrm{peak}}$ by ~1–5 mL^.^kg^-1.^min^-1^. Many of the traditional LT methods were not valid and a newly developed Modified D_max_ method derived from GXT_4_ provided the most valid estimate of the MLSS (MD = 1.1 W; ICC = 0.96).

**Conclusion:**

The data highlight how GXT protocol design and data analysis influence the determination of both ${\dot{V}O}_{2\mathrm{peak}}$ and LT. It is also apparent that ${\dot{V}O}_{2\max}$ and LT cannot be determined in a single GXT, even with the inclusion of a VEB.

## Introduction

Sampling of expired gas and blood data during a graded exercise test (GXT) to exhaustion permits identification of the gas exchange threshold (GET), the respiratory compensation point (RCP), the lactate threshold (LT), and maximal oxygen uptake (${\dot{V}O}_{2\max}$). These indices can distinguish cardiorespiratory fitness, and demarcate the domains of exercise [1, 2] that can be used to prescribe exercise and to optimize training stimuli [3–6]. However, despite the popularity of these indices, the methods used to determine them can differ substantially and there has been little systematic investigation of their validity [7–9].

The recommended duration of a GXT to assess ${\dot{V}O}_{2\max}$ is 8 to 12 minutes [10–13]. However, there is little consensus on an appropriate GXT protocol design, including duration, stage length, or number of stages, needed to establish the LT. A stage length of at least 3 minutes has been recommended [13], although an 8-minute stage length has also been suggested for blood lactate concentrations to stabilize [14]. The number of stages and GXT duration will depend on the starting intensity and power increments. Power is typically increased identically [15], regardless of sex or fitness, leading to a heterogenous GXT duration and number of stages completed [16]. A customized approach to LT testing has been recommended to ensure a more homogenous GXT duration [17].

More than 25 methods have been proposed to calculate the LT [18]; these include the power preceding a rise in blood lactate concentration of more than 0.5, 1.0 or 1.5 mmol^.^L^-1^ from baseline [19], the onset of a fixed blood lactate accumulation (OBLA) ranging from 2.0 to 4.0 mmol^.^L^-1^ [20, 21], or the use of curve fitting procedures such as the D_max_ or modified D_max_ methods (ModD_max_) [22, 23]. However, many of these ‘accepted’ methods are influenced by GXT protocol design [8, 24] and their underlying validity has not been reported.

Assessing the validity of a measurement requires comparison with a criterion measure. The maximal lactate steady state (MLSS) represents the highest intensity where blood lactate appearance and disappearance is in equilibrium and where energy demand is adequately met by oxidative phosphorylation [25]. Exercise performed above the MLSS results in accelerated blood lactate appearance and it has therefore been suggested as an appropriate criterion measure for the LT [25, 26]. The primary advantages of the MLSS test include its independence of participant effort, it’s submaximal and is reliable [27]. However, the disadvantage is the necessity of multiple laboratory visits and that it yields only one index of performance.

${\dot{V}O}_{2\max}$ is considered the “gold standard” for assessing cardiorespiratory fitness [28] and the highest recorded ${\dot{V}O}_{2}$ from a GXT is often accepted as the ${\dot{V}O}_{2\max}$ [10]. Establishing the LT requires a GXT that typically exceeds 20 minutes [13]; however, in these instances the highest ${\dot{V}O}_{2}$ may underestimate the ${\dot{V}O}_{2\max}$ [12] and is termed ${\dot{V}O}_{2\mathrm{peak}}$. Recently, the use of a verification exhaustive bout (VEB) has been recommended to confirm the ${\dot{V}O}_{2\max}$. However, it is unknown if a VEB performed after a longer duration GXT provides a valid estimate of ${\dot{V}O}_{2\max}$.

The aim of this study was to determine the validity of the LT and ${\dot{V}O}_{2\max}$ derived from a single visit GXT. We hypothesized that our results would yield one or more GXT stage length and LT calculation method combination that provides a valid estimation of the criterion measure of the LT (i.e., MLSS). We also hypothesized the highest ${\dot{V}O}_{2}$ measured during longer duration GXTs would underestimate ${\dot{V}O}_{2\max}$ and that the highest ${\dot{V}O}_{2}$ value measured during each VEB would be similar to the ${\dot{V}O}_{2\mathrm{peak}}$ measured during the 8- to 12-minute GXT.

## Materials and methods

### Ethical approval

All procedures were performed in accordance with the ethical standards of the institutional and/or national research committee, and with the 1964 Helsinki declaration and its later amendments or comparable ethical standards.

### Participants/Experimental design

Seventeen trained male cyclists (${\dot{V}O}_{2\max}$ 62.1 ± 5.8 mL^.^kg^-1.^min^-1^, age 36.2 ± 7.4 years, body mass index (BMI): 24.1 ± 2.0 kg^.^m^-2^) volunteered for this study which required 7 to 10 visits to the laboratory. Informed consent was obtained from all individual participants included in the study.

Visit one included risk stratification using the American College of Sports Medicine Risk Stratification guidelines [29], written informed consent, self-reported physical activity rating (PA-R) [30], measurement of height and body mass, and completion of a cycling GXT with 1-minute stages (GXT_1_) followed by a VEB. The remaining visits consisted of four cycling GXTs with varying stage length (3-, 4-, 7- and 10-min stages) and a series of 30-min constant power bouts to establish the MLSS. The GXTs and constant power bouts were performed in an alternating order and the order of the GXTs was randomised. Prior to each GXT and the constant power bouts a 5-min warm up was administered at a self-selected power followed by 5 min of passive rest. Participants performed each test at their preferred cadence determined during the initial visit. Antecubital venous blood (1.0 mL) was sampled during all visits (excluding GXT_1_) at rest, and at the end of every stage during the GXTs or every 5 min during the constant power exercise bouts. All participants self-reported abstaining from the consumption of alcohol and caffeine or engaging in heavy exercise 24 h prior to each visit. Participants were given at least 48 h between visits and all tests were completed within 6 weeks. The Victoria University Human Research Ethics Committee approved all procedures (HRE 017–035).

### Equipment/Instruments

All exercise testing was conducted using an electronically-braked cycle ergometer (Lode Excalibur v2.0, The Netherlands). A metabolic analyser (Quark Cardiopulmonary Exercise Testing, Cosmed, Italy) was used to assess oxygen uptake (${\dot{V}O}_{2}$) on a breath-by-breath basis, and heart rate was measured throughout all tests. Antecubital venous blood was analysed using a blood lactate analyser (YSI 2300 STAT Plus, YSI, USA).

### GXTs with verification exhaustive bout

Demographic data, PA-R, and measurements of height and body mass were used to estimate ${\dot{V}O}_{2\max}$ [31] and maximum power output ${\dot{W}}_{\max}$ [30, 32].

Est.VO2max=56.363+(1.921xPA−R)–(0.381xAGE)–(0.754xBMI)+(10.987xSEX,1=MALE,0=FEMALE) Eq 1

$$\dot{W}max=\{[(VO2max–7)\;x\;BM]/1.8\}/6.12$$ Eq 2

Where ${\dot{V}O}_{2\max}$ is expressed in millilitres per kilogram per minute, BMI is in kg^.^m^-2^, and ${\dot{W}}_{\max}$ is in Watts.

A custom GXT protocol with a desired time limit of 10 min was then designed for each particpant using: ${\dot{W}}_{\max}/10$ min = 1-min intensities (W^.^min^-1^). Additional customized protocols were designed for each of the remaining GXTs based on a percentage of the measured ${\dot{W}}_{\max}$ from GXT_1_. The predicted ${\dot{W}}_{\max}$ was 80%, 77%, 72% and 70% for GXT_3,_ GXT_4_, GXT_7_, and GXT_10_, respectively. The target number of stages for each participant was nine; the initial stage and subsequent stages of the remaining GXTs were determined using the following equations:

$$Stage\;1\;Power=Predicted\;\dot{W}max\;*\;0.25$$ Eq 3

$$Subsequent\;power\;increments=(Predicted\;\dot{W}max–Stage\;1)/8)$$ Eq 4

where stage 1 power and predicted ${\dot{W}}_{\max}$ subsequent power increments are expressed in Watts.

A 5-min recovery was administered after each GXT, followed by a VEB performed at 90% of ${\dot{W}}_{\max}$ measured from GXT_1_ to measure the highest measured ${\dot{V}O}_{2}$ measure (${\dot{V}O}_{2\mathrm{peak}}$) [17].

### Constant power exercise bouts to establish the maximal lactate steady state

The power associated with the respiratory compensation point (RCP) from GXT_1_ was used in a regression equation (Eq 5) to estimate the MLSS (RCP_MLSS_) and the first constant power exercise [33]. The RCP was determined as the average of the power output associated with: 1) the break point in ventilation relative to expired carbon dioxide (${\dot{V}}_{E}/{\dot{V}\mathrm{CO}}_{2}$), 2) second break point in ${\dot{V}}_{E}$ and 3) the fall in end-tidal carbon dioxide (P_ET_CO_2_) after an apparent steady state [34–36].

EstimatedMLSS(RCPMLSS)=23.329+(0.79127xRCP) Eq 5

where the RCP_MLSS_ and RCP are expressed in Watts

Participants performed 3 min of baseline cycling at 20 W prior to each constant power bout. The MLSS was established as the highest intensity where blood lactate increased <1.0 mmol^.^L^-1^ from the 10^th^ to the 30^th^ minute [26]. If the blood lactate concentration increased >1.0 mmol^.^L^-1^ the power was decreased by 3%, otherwise the power was increased by 3% [27]. This process continued until the MLSS was obtained.

### LT and respiratory compensation point calculations

**Fig 1:**
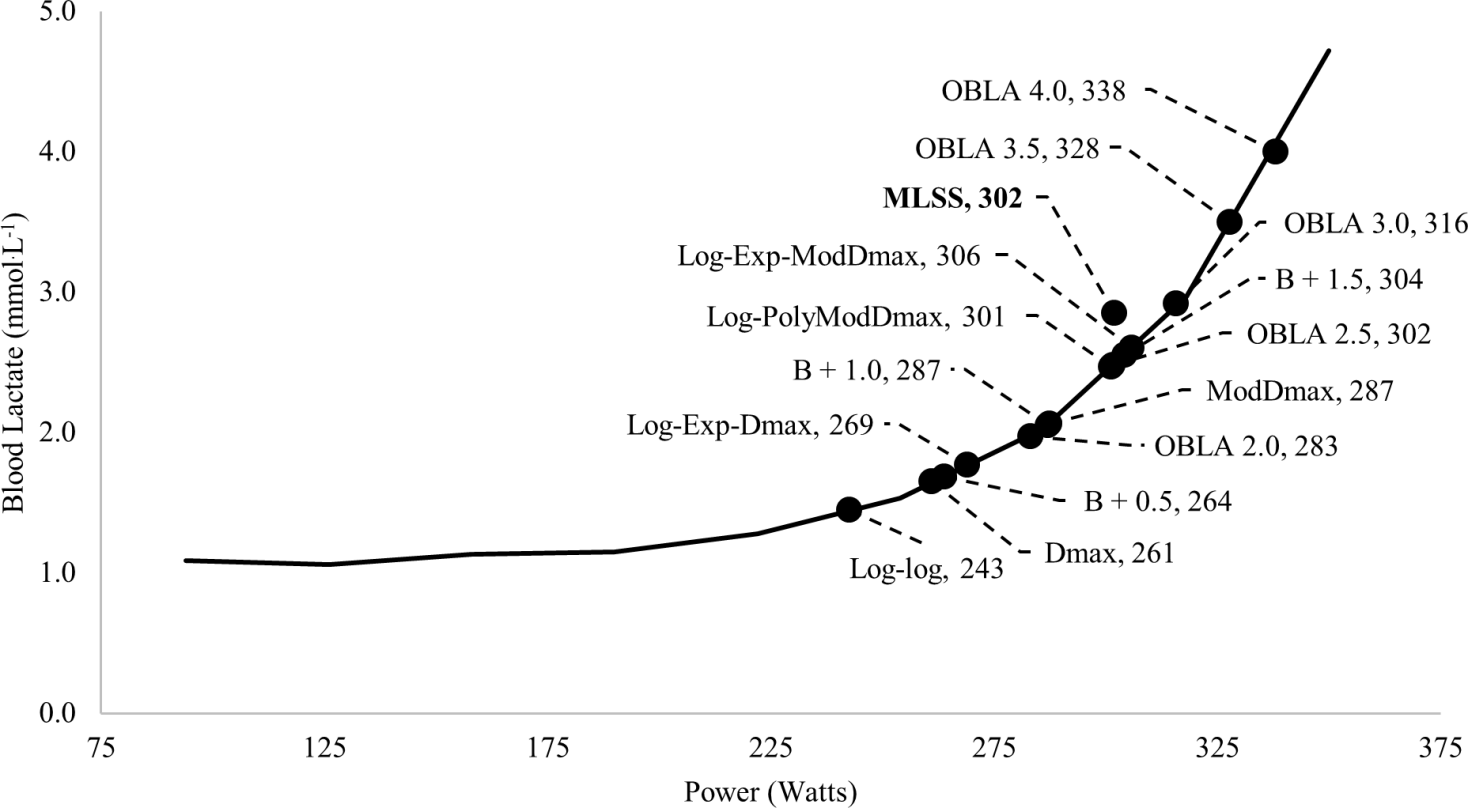
Representative blood lactate curve with 14 LTs calculated from GXT_4_ (participant #9). The power of the MLSS was 302 W and the blood lactate concentration was 2.85 mmol^.^L^-1^. Log-log = power at the intersection of two linear lines with the lowest residual sum of squares; log = using the log-log method as the point of the initial data point when calculating the D_max_ or Modified D_max_; poly = Modified D_max_ method calculated using a third order polynomial regression equation; exp = Modified D_max_ method calculated using a constant plus exponential regression equation; OBLA = onset of blood lactate accumulation; B + absolute value = the intensity where blood lactate increases above baseline.

The LTs were calculated from GXT_3,4,7 and 10_ using 14 methods (4 GXTs * 14 LTs = 56 LTs in total), and the RCP and the RCP_MLSS_ were also calculated from GXT_1_ (56 LTs + RCP and RCP_MLSS_ = 58 total estimates) (Fig 1):

1. Log-log: The lactate curve was divided into two segments and the intersection point of the two lines with the lowest residuals sum of squares was taken as the LT [37].
2. OBLA value of 2.0, 2.5, 3.0, 3.5, or 4.0 mmol^.^L^-1^ [1, 24, 38].
3. Baseline + absolute value(s) (B + mmol^.^L^-1^): The intensity at which blood lactate concentration increased 0.5, 1.0 or 1.5 mmol^.^L^-1^ above baseline value(s) [39, 40].
4. D_max_: The point on the third order polynomial regression curve that yielded the maximum perpendicular distance to the straight line formed by the two end points of the curve [23].
5. Modified D_max_ (ModD_max_): The intensity at the point on the third order polynomial regression curve that yielded the maximal perpendicular distance to the straight line formed by the point preceding the first rise in blood lactate concentration of >0.4 mmol^.^L^-1^ lactate and the final lactate point [22].
6. Exponential D_max_ (Exp-D_max_): The point on the exponential plus-constant regression curve that yielded the maximum perpendicular distance to the straight line formed by the two end points of the curve [41, 42].
7. Log-log Modified D_max_ (Log-Poly-ModD_max_): The intensity at the point on the third order polynomial regression curve that yielded the maximal perpendicular distance to the straight line formed by the intensity associated with the log-log LT and the final lactate point.
8. Log-log Exponential Modified D_max_ method (Log-Exp-ModD_max_): The intensity at the point on the exponential plus-constant regression curve that yielded the maximal perpendicular distance to the straight line formed by the intensity associated with the log-log LT and the final lactate point.
9. RCP: refer to Constant Power Exercise Bouts to Establish the Maximal Lactate Steady State method section.
10. The estimated MLSS was based on a regression equation based on the RCP from GXT_1_ (RCP_MLSS_) (Eq 5).

## Data analysis

Breath-by-breath data were edited individually with values greater than three standard deviations from the mean excluded [43]. The data was interpolated on a second-by-second basis and averaged into 5- and 30-s bins [44, 45]. The highest measured ${\dot{V}O}_{2}$ value from every GXT and VEB was determined as the highest 20-s rolling average. The ${\dot{V}O}_{2\max}$ was computed as the highest ${\dot{V}O}_{2}$ measured from any GXT or VEB. The ${\dot{V}O}_{2\mathrm{peak}}$ for each GXT was defined as the highest measured ${\dot{V}O}_{2}$ from either the GXT or the subsequent VEB.

The ${\dot{W}}_{\max}$ for every GXT was determined as the power from the last completed stage plus the time completed in the subsequent stage multiplied by the slope (Eq 6). The ${\dot{V}O}_{2}$ response at the MLSS was determined by the average ${\dot{V}O}_{2}$ value during the last two minutes of the 30-minute constant power bout.

$$\dot{W}max=Power\;of\;Last\;Stage\;(W)+[slope\;(W.s-1)*time\;(sec.)]$$ Eq 6

Calculated LTs were excluded if the mean difference between the MLSS and calculated LT was greater than the error of the measurement of the MLSS [coefficient of the variation (CV%) = 3%, 7.9 W] [27], the effect size (ES) was greater than 0.2, or the Pearson Product moment correlation coefficient (r) was less than 0.90. Using these criteria, 10 of the 56 LTs and the RCP_MLSS_ (Eq 5) were included in the analysis (Table 1).

**Table 1:** The mean ± standard deviation (SD) of the 14 lactate thresholds calculated from the 4 prolonged graded exercise tests (i.e., GXT_3_, GXT_4_, GXT_7_ and GXT_10_), and the respiratory compensation point (RCP) and the maximal lactate steady state (MLSS) estimated from the RCP (RCP_MLSS_) calculated from GXT_1_.

		GXT_3_	GXT_4_	GXT_7_	GXT_10_
**Log-log LT**	**Mean SD (W)**	211 ± 43	202 ± 38	200 ± 40	196 ± 41
** **	**MD (W)**	53.1	62.8	64.8	68.3
	**r**	0.84	0.89	0.87	0.78
** **	**ES**	1.28	1.63	1.62	1.70
**OBLA 2.0**	**Mean SD (W)**	262 ± 40	249 ± 39	247 ± 39	245 ± 37
** **	**MD (W)**	2.1	15.1	17.3	19.6
** **	**r**	0.86	0.94	0.94	0.93
	**ES**	-0.05	-0.38	-0.44	-0.50
**OBLA 2.5**	**Mean SD (W)**	276 ± 42	**262** ± **40**	**258** ± **40**	255 ± 38
** **	**MD (W)**	-11.9	**2.0**	**6.7**	9.2
** **	**r**	0.89	**0.95**	**0.94**	0.93
	**ES**	0.30	**-0.05**	**-0.17**	-0.23
**OBLA 3.0**	**Mean SD (W)**	288 ± 43	273 ± 41	**267** ± **41**	**264** ± **39**
** **	**MD (W)**	-23.2	-8.8	**-2.2**	**0.4**
	**r**	0.90	0.96	**0.95**	**0.93**
** **	**ES**	0.59	0.22	**0.06**	**-0.01**
**OBLA 3.5**	**Mean SD (W)**	297 ± 45	282 ± 41	274 ± 41	**272** ± **40**
** **	**MD (W)**	-32.8	-18.1	-10.0	**-7.3**
	**r**	0.91	0.96	0.95	**0.93**
** **	**ES**	0.83	0.46	0.25	**0.19**
**OBLA 4.0**	**Mean SD (W)**	306 ± 46	291 ± 42	281 ± 42	279 ± 41
** **	**MD (W)**	-41.3	-26.3	-16.8	-14.2
	**r**	0.91	0.97	0.95	0.93
** **	**ES**	1.05	0.67	0.43	0.36
**Baseline + 0.5**	**Mean SD (W)**	235 ± 38	229 ± 40	228 ± 41	225 ± 37
** **	**MD (W)**	29.4	35.6	36.6	39.5
** **	**r**	0.74	0.81	0.83	0.82
	**ES**	-0.75	-0.90	-0.93	-1.00
**Baseline + 1.0**	**Mean SD (W)**	255 ± 39	239 ± 40	236 ± 39	235 ± 39
** **	**MD (W)**	9.5	25.3	27.9	29.1
	**r**	0.88	0.92	0.93	0.91
** **	**ES**	-0.24	-0.64	-0.71	-0.74
**Baseline + 1.5**	**Mean SD (W)**	**270** ± **41**	254 ± 41	250 ± 39	248 ± 39
** **	**MD (W)**	**-6.0**	10.1	14.7	16.8
** **	**r**	**0.90**	0.94	0.94	0.92
	**ES**	**0.15**	-0.26	-0.37	-0.43
**Dmax**	**Mean SD (W)**	246 ± 34	232 ± 36	223 ± 31	216 ± 33
** **	**MD (W)**	18.6	31.9	41.6	48.8
** **	**r**	0.94	0.97	0.96	0.95
	**ES**	-0.47	-0.81	-1.06	-1.24
**Modified Dmax**	**Mean SD (W)**	278 ± 37	**267** ± **39**	255 ± 40	248 ± 37
** **	**MD (W)**	-13.2	**-2.9**	9.7	15.9
** **	**r**	0.90	**0.91**	0.93	0.92
	**ES**	0.33	**0.07**	-0.25	-0.40
**Log-Poly-MDmax**	**Mean SD (W)**	280 ± 42	**265** ± **42**	255 ± 39	248 ± 40
** **	**MD (W)**	-15.5	**-1.1**	9.5	16.5
	**r**	0.94	**0.96**	0.96	0.92
** **	**ES**	0.39	**0.03**	-0.24	-0.42
**Exp-Dmax**	**Mean SD (W)**	256 ± 35	243 ± 36	234 ± 34	228 ± 35
** **	**MD (W)**	8.0	21.8	30.8	36.8
	**r**	0.92	0.97	0.96	0.94
** **	**ES**	-0.20	-0.55	-0.78	-0.93
**Log-Exp-MDmax**	**Mean SD (W)**	286 ± 42	**271** ± **42**	**260** ± **39**	253 ± 40
	**MD (W)**	-21.7	**-7.0**	**4.3**	11.1
	**r**	0.94	**0.97**	**0.96**	0.93
	**ES**	0.55	**0.18**	**-0.11**	-0.28
		**GXT**_**1**_			
**RCP**_**MLSS**_	**Mean SD (W)**	**271 ± 39**			
	**MD (W)**	**-6.71**			
	**r**	**0.92**			
	**ES**	**-0.17**			
**RCP**	**Mean SD (W)**	315 ± 40			
	**MD (W)**	-50.4			
	**r**	0.91			
	**ES**	1.27			

Also shown is the mean difference (MD), the Pearson product moment correlation (r) and effect size (ES) of the difference when compared with the MLSS. (log = using the log-log method as the point of the initial data point when calculating the D_max_ or Modified D_max_; poly = Modified D_max_ method calculated using a third order polynomial regression equation; exp = Modified D_max_ method calculated using a constant plus exponential regression equation; OBLA = onset of blood lactate accumulation, B + = baseline lactate value plus an absolute lactate value). **Bold** represents the LT that met the three criteria for inclusion in our final analysis: mean difference less than 7.9 Watts, Pearson moment product correlation >0.90, and a less than trivial ES difference from the MLSS (ES <0.2)

## Statistical analysis

A one-way analysis of variance with repeated measures was used to assess significant differences between the MLSS and the calculated LTs. Agreement between the MLSS and the calculated LTs was evaluated using a two-way mixed intraclass correlation coefficient (ICC), standard error of the measurement (SEM), Lin’s concordance correlation coefficient (p_c_) [46], Bland-Altman plots [47], (r), CV% [48, 49] and a magnitude-based inference approach involving standardised differences (ED) [50, 51]. Differences between ${\dot{V}O}_{2\mathrm{peak}}$ values measured during each GXT were assessed using ES, p-values, and the CV%. Agreement between ${\dot{V}O}_{2}$ measured during each GXT and subsequent VEB was evaluated using intraclass calculation coefficient (ICC), SEM, and CV% [49]. Descriptive statistics are reported as the mean ± SD. Alpha was set to P ≤ 0.05.

## Results

### MLSS

The power associated with the MLSS was 264 ± 39 W, and the blood lactate concentrations at the 10^th^ and 30^th^ min were 2.8 ± 0.8 and 3.3 ± 0.8 mmol^.^L^-1^, respectively. The blood lactate values at 3% above the MLSS (272 ± 41 W) at the 10^th^ and 30^th^ min were 3.6 ± 0.8 and 5.0 ± 0.9 mmol^.^L^-1^, respectively. The ${\dot{V}O}_{2}$ at the MLSS was 81.4 ± 4.7% of ${\dot{V}O}_{2\max}$ (3892 ± 441 mL^.^min^-1^; 50.5 ± 4.0 mL^.^kg^-1.^min^-1^). For each GXT the ${\dot{V}O}_{2}$ at the MLSS and the power at the MLSS are shown in Table 2.

**Table 2:** Mean, standard deviation, and range of the ${\dot{V}O}_{2}$ and power associated with the maximal lactate steady state (MLSS) expressed as a percentage of the maximal power (${\dot{W}}_{\max}$) and ${\dot{V}O}_{2\mathrm{peak}}$ measured during each GXT. Note: The ${\dot{V}O}_{2}$ at the MLSS was 81.4 ± 4.7% of the ${\dot{V}O}_{2\max}$. (Defined as the highest measured ${\dot{V}O}_{2}$ during any GXT).

	GXT_1_	GXT_3_	GXT_4_	GXT_7_	GXT_10_
${\dot{V}O}_{2}$ **at MLSS** **(% of** ${\dot{V}O}_{2\mathrm{peak}}$**)**	83.0 ± 4.5 [75.5–90.7]	84.7 ± 4.7 [76.6–91.9]	86.1 ± 5.9 [73.9–94.2]	88.4 ± 6.0 [77.4–103.2]	90.2 ± 5.3 [78.7–99.9]
**Power at MLSS** **(% of** ${\dot{W}}_{\max}$**)**	62.9 ± 3.9 [56.8–71.7]	78.4 ± 4.3 [69.8–84.4]	82.4 ± 3.6 [73.7–88.8]	87.3 ± 4.4 [79.8–96.0]	89.6 ± 4.7 [81.6–98.1]

### Validity of LT estimates

**Fig 2:**
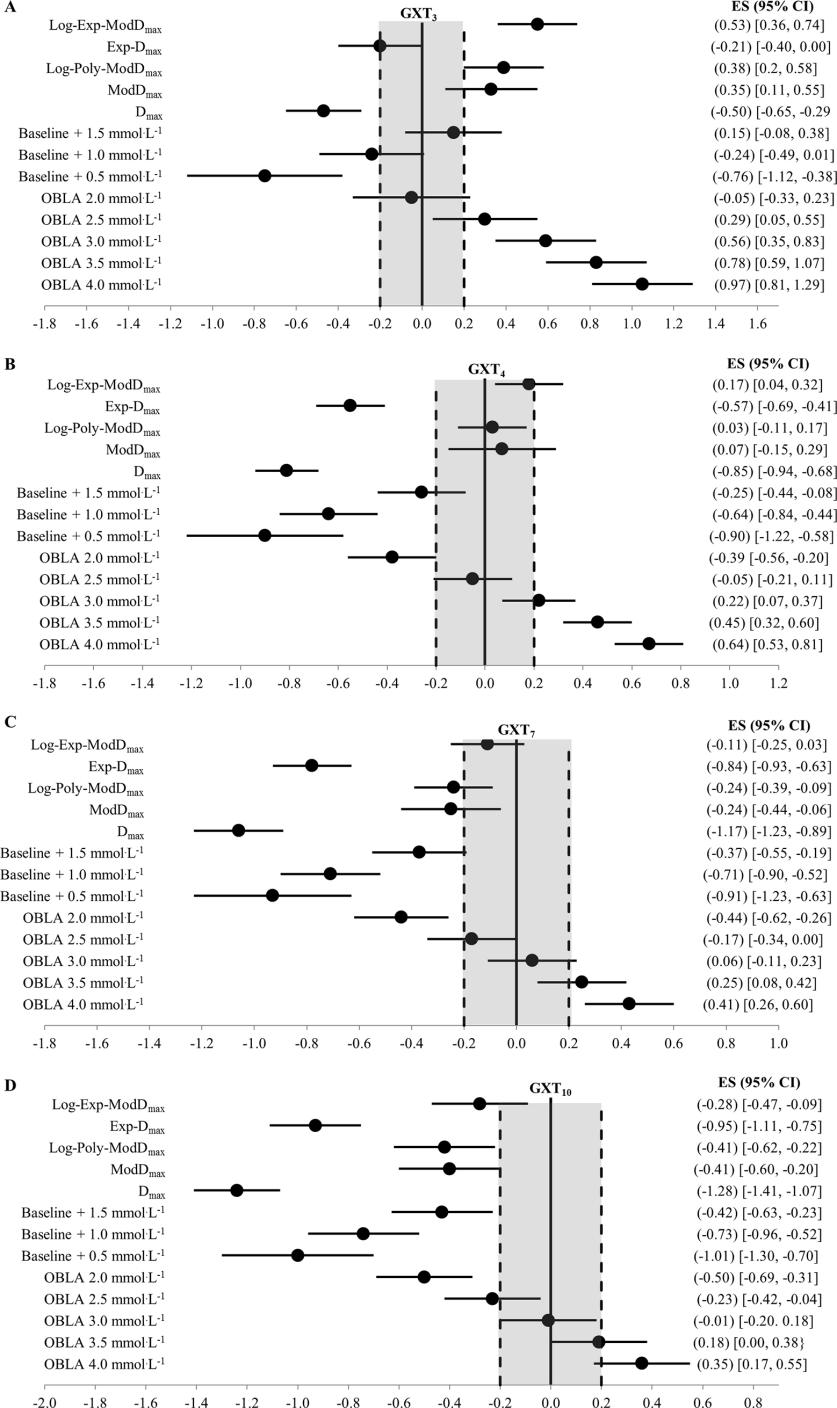
(A-D) Forrest Plots of the difference (ES ± 95% CI) between the MLSS and the power calculated from the 13 lactate thresholds derived from (A) GXT_3_, (B) GXT_4_, (C) GXT_7_ and (D) GXT_10_ (52 in total and excluding log-log). The solid vertical bar represents no difference from the MLSS and the dashed vertical bars represents the threshold between a trivial and small difference (ES = 0.2) established by Cohen (50) and Hopkins (49). log = using the log-log method as the initial data point when calculating the D_max_ or Modified D_max_; poly = Modified D_max_ method calculated using a third order polynomial regression equation; exp = Modified D_max_ method calculated using a constant plus exponential regression equation; OBLA = onset of blood lactate accumulation.

**Fig 3:**
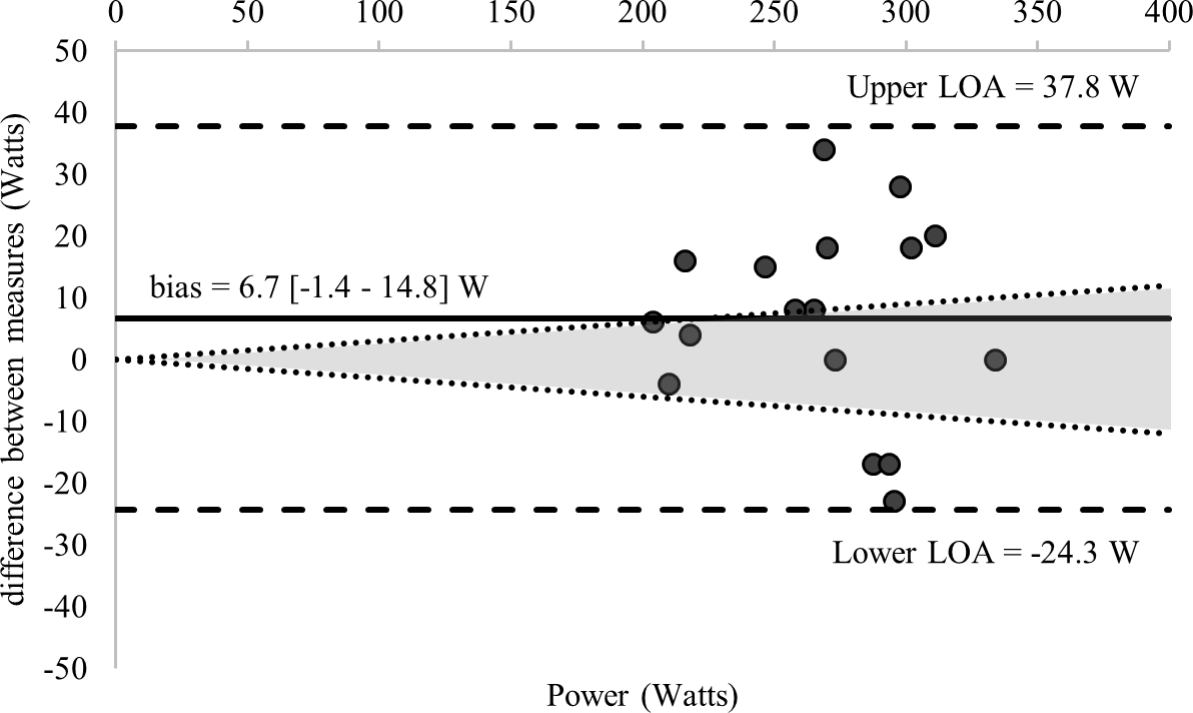
Bland-Altman plots displaying agreement between measures of the power associated with the RCP regression equation (RCP_MLSS_) calculated from GXT_1_ and the MLSS. The differences between measures (y-axis) are plotted as a function of the mean of the two measures (x-axis) in power (Watts). The horizontal solid line represents the mean difference between the two measures (i.e., bias). The two horizontal dashed lines represent the limits of agreement (1.96 x standard deviation of the mean difference between the estimated lactate threshold via the RCP_MLSS_ and the maximal lactate steady state). The dotted diagonal lines represent the boundaries of the 95% CI for MLSS reliability (CV = 3.0%; 95%; CI = 3.8%) calculated from Hauser et al., 2014) (RCP = respiratory compensation point).

**Fig 4:**
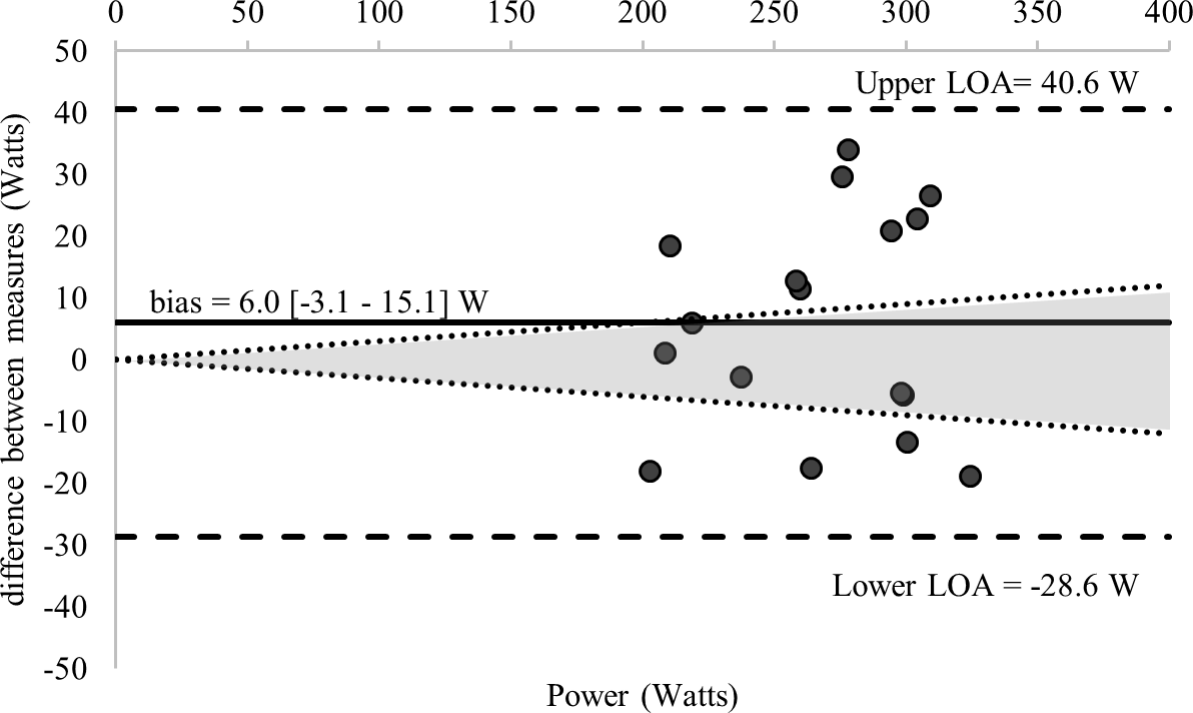
Bland-Altman plots displaying agreement between measures of the power associated with the baseline plus 1.5 mmol^.^L^-1^ calculated from GXT_3_ and the MLSS. The differences between measures (y-axis) are plotted as a function of the mean of the two measures (x-axis) in power (Watts). The horizontal solid line represents the mean difference between the two measures (i.e., bias). The two horizontal dashed lines represent the limits of agreement (1.96 x standard deviation of the mean difference between the lactate threshold and the maximal lactate steady state). The dotted diagonal lines represent the boundaries of the 95% CI for MLSS reliability (CV = 3.0%; 95%; CI = 3.8%) calculated from Hauser et al., 2014).

**Fig 5:**
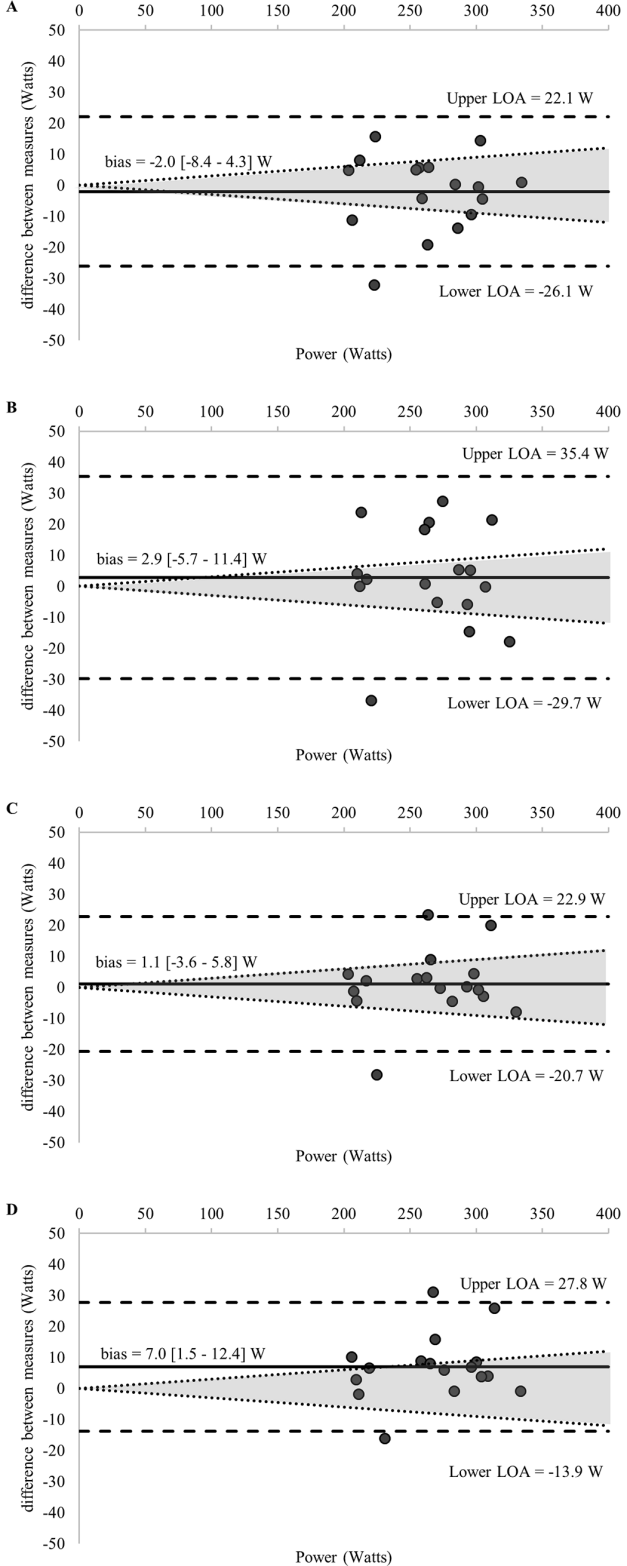
(A-D) Bland-Altman plots displaying agreement between measures of the power associated with the (A) OBLA 2.5 mmol^.^L^-1^, (B) Modified D_max_, (C) Log-Poly-Modified D_max_, (D) Log-Exp-Modified D_max_ calculated from **GXT**_**4**_ and the MLSS. The differences between measures (y-axis) are plotted as a function of the mean of the two measures (x-axis) in power (Watts). The horizontal solid line represents the mean difference between the two measures (i.e., bias). The two horizontal dashed lines represent the limits of agreement (1.96 x standard deviation of the mean difference between the lactate threshold and the maximal lactate steady state). The dotted diagonal lines represent the boundaries of the 95% CI for MLSS reliability (CV = 3.0%; 95%; CI = 3.8%) calculated from Hauser et al., 2014) (log = Modified D_max_ method using the log-log method as the point of the initial lactate point; poly = Modified D_max_ method calculated using a third order polynomial regression equation; exp = Modified D_max_ method calculated using a constant plus exponential regression equation; OBLA = onset of blood lactate accumulation.).

**Fig 6:**
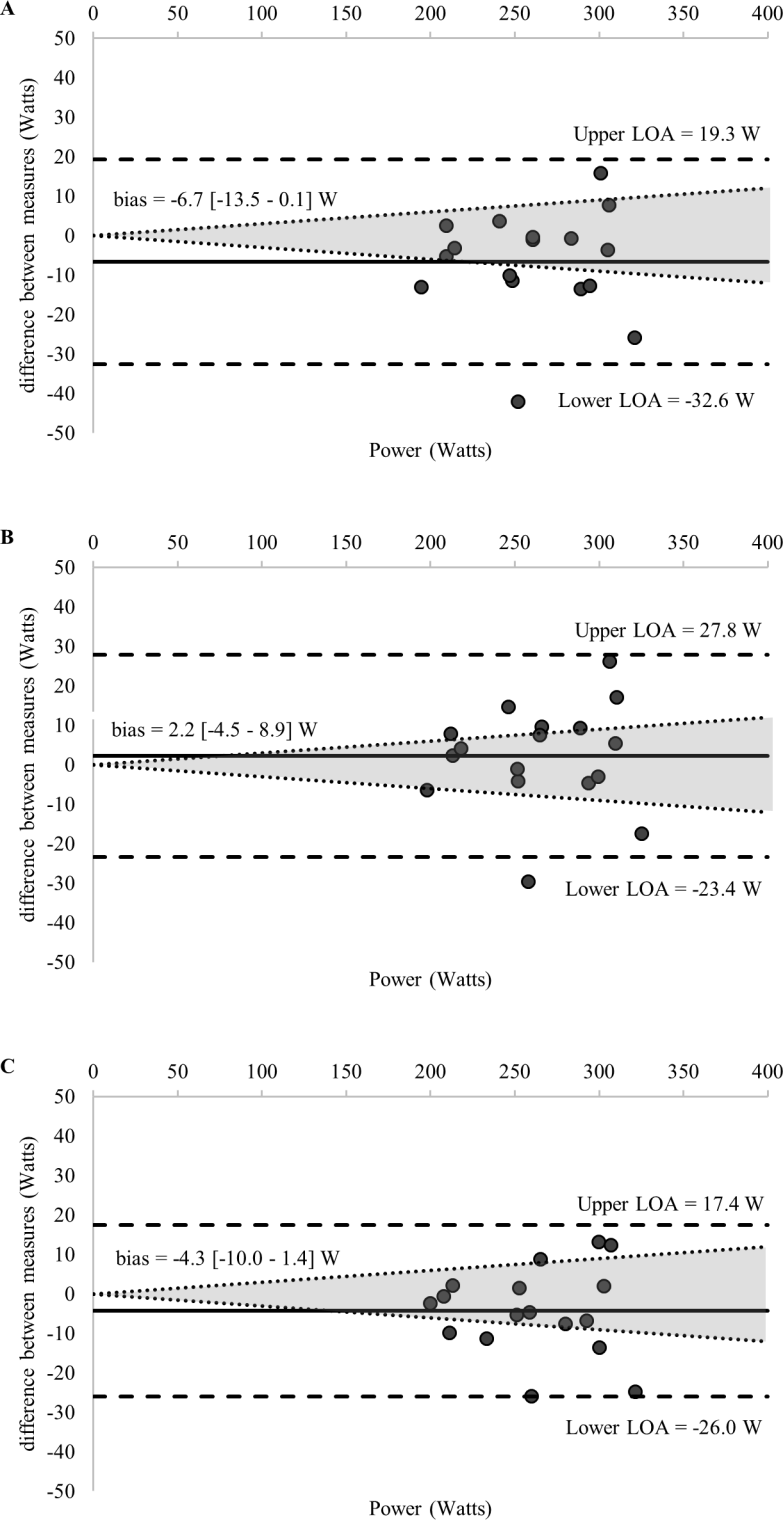
(A-C) Bland-Altman plots displaying agreement between measures of the power associated with the (A) OBLA 2.5 mmol^.^L^-1^ (GXT_7_), (B) OBLA 3.0 mmol^.^L^-1^ (GXT_7_), (C) Log-Exp-Modified D_max_ calculated from **GXT**_**7**_ and the MLSS. The differences between measures (y-axis) are plotted as a function of the mean of the two measures (x-axis) in power (Watts). The horizontal solid line represents the mean difference between the two measures (i.e., bias). The two horizontal dashed lines represent the limits of agreement (1.96 x standard deviation of the mean difference between the lactate threshold and the maximal lactate steady state). The dotted diagonal lines represent the boundaries of the 95% CI for MLSS reliability (CV = 3.0%; 95%; CI = 3.8%) calculated from Hauser et al., 2014) (log = Modified D_max_ method using the log-log method as the point of the initial lactate point; exp = Modified D_max_ method calculated using a constant plus exponential regression equation; OBLA = onset of blood lactate accumulation.).

**Fig 7:**
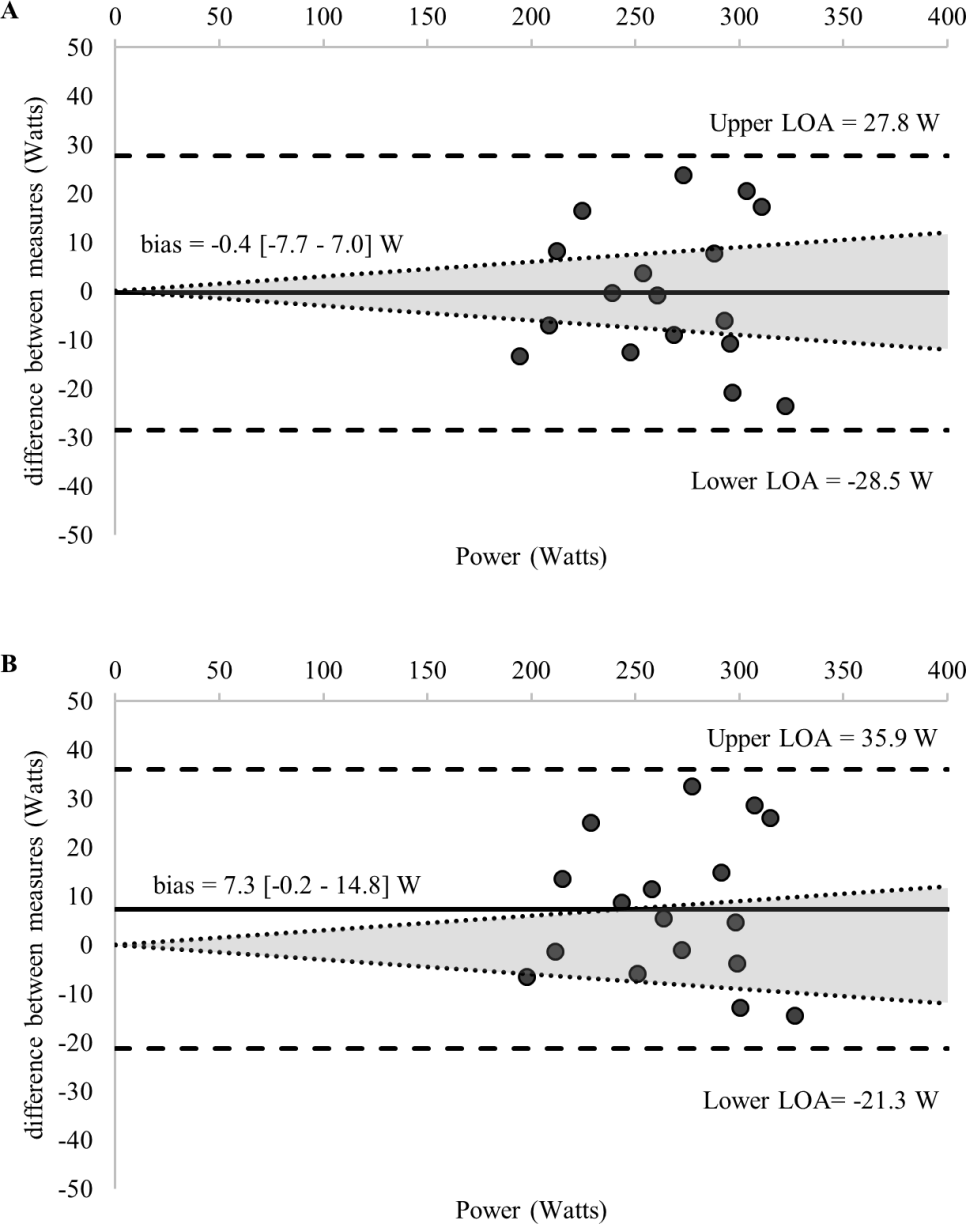
(A-B) Bland-Altman plots displaying agreement between measures of the power associated with the (A) OBLA 3.0 mmol^.^L^-1^, (B) OBLA 3.5 mmol^.^L^-1^ calculated from **GXT**_**10**_ and the MLSS. The differences between measures (y-axis) are plotted as a function of the mean of the two measures (x-axis) in power (Watts). The horizontal solid line represents the mean difference between the two measures (i.e., bias). The two horizontal dashed lines represent the limits of agreement (1.96 x standard deviation of the mean difference between the lactate threshold and the maximal lactate steady state). The dotted diagonal lines represent the boundaries of the 95% CI for MLSS reliability (CV = 3.0%; 95%; CI = 3.8%) calculated from Hauser et al., 2014) (OBLA = onset of blood lactate accumulation.).

Comparisons of the 58 estimations of the MLSS and the calculated MLSS are detailed in Table 1. Fig 2 displays the standardized difference of the 13 LTs calculated for each GXT (52 in total) and the MLSS (all log-log methods were excluded given an ES > 1.0). Ten of the calculated LTs and the RCP_MLSS_ met our inclusion criteria for final analysis—detailed comparisons with the MLSS are provided in Table 3 and Fig 3. Figs 3–7 shows Bland-Altman plots of the 11 estimations included in our analysis; the newly developed ModD_max_ LT calculations (Fig 5 Panel C and D; Fig 6 Panel C) had the lowest limits of agreement with the MLSS. The log-log polynomial modified D_max_ (Log-Poly-ModD_max_) method derived from GXT_4_ provided the best estimation of the MLSS (Fig 5 Panel C). There was an inverse relationship between the power calculated for each of the 14 LTs and stage length (Tables 1 and 4).

**Table 3:** Mean ± standard deviation, mean difference (MD), intraclass correlation coefficient (ICC), Lin’s concordance correlation coefficient (ρ_c_), standard error of the measurement (SEM), effect size (ES) with 95% confidence limits, and coefficient of the variation (%CV) between the maximal lactate steady state (MLSS) and the eleven thresholds included in our analysis. (RCP_MLSS_ = MLSS estimate based on the respiratory compensation point; log = Modified D_max_ method using the log-log method as the point of the initial lactate point; poly = Modified D_max_ method calculated using a third order polynomial regression equation; exp = Modified D_max_ method calculated using a constant plus exponential regression equation; OBLA = onset of blood lactate accumulation).

		Mean ± SD (W)	MD (W)	ICC [95% CI]	ρ_c_	SEM [95% CI] (W)	ES [95% CI]	CV [95% CI] (%)
	**MLSS**	**264 ± 39**						
**GXT**_**1**_	**RCP**_**MLSS**_	271 ± 39	6.7	0.92 [0.78–0.97]	0.90	11.2 [8.3–17.0]	0.17 [-0.04–0.38]	6.0 [4.4–9.4]
**GXT**_**3**_	**Baseline + 1.5 mmol**^.^**L**^**-1**^	270 ± 41	6.0	0.90 [0.75–0.97]	0.90	12.5 [9.3–19.0]	0.15 [-0.08–0.38]	6.6 [4.9–10.4]
**GXT**_**4**_	**OBLA 2.5 mmol**^.^**L**^**-1**^	262 ± 40	-2.0	0.95 [0.87–0.98]	0.95	8.7 [6.5–13.2]	-0.05 [-0.21–0.11]	5.3 [3.9–8.4]
	**Modified D**_**max**_	267 ± 39	2.9	0.91 [0.76–0.98]	0.90	11.7 [8.7–17.9]	0.07 [-0.15–0.29]	7.0 [5.1–11.0]
	**Log-Poly-MD**_**max**_	265 ± 42	1.1	0.96 [0.90–0.99]	0.96	7.9 [5.8–12.0]	0.03 [-0.11–0.17]	4.4 [3.2–6.9]
	**Log-Exp-MD**_**max**_	271 ± 42	7.0	0.97 [0.91–0.99]	0.95	7.5 [5.6–11.4]	0.18 [0.04–0.32]	4.1 [3.0–6.3]
**GXT**_**7**_	**OBLA 2.5 mmol**^.^**L**^**-1**^	258 ± 41	-6.7	0.94 [0.85–0.98]	0.93	9.4 [7.0–14.3]	-0.17 [-0.34–0.00]	4.9 [3.6–7.7]
	**OBLA 3.0 mmol**^.^**L**^**-1**^	267 ± 41	2.2	0.95 [0.86–0.98[	0.95	9.2 [6.9–14.1]	0.06 [-0.11–0.23]	5.1 [3.7–8.0]
	**Log-Exp-MD**_**max**_	260 ± 39	-4.3	0.96 [0.89–0.99]	0.95	7.8 [5.8–11.9]	-0.11 [-0.25–0.03]	4.1 [3.0–6.4]
**GXT**_**10**_	**OBLA 3.0 mmol**^.^**L**^**-1**^	264 ± 39	-0.4	0.93 [0.82–0.98]	0.93	10.2 [7.6–15.5]	-0.01 [-0.20–0.18]	5.5 [4.0–8.6]
	**OBLA 3.5 mmol**^.^**L**^**-1**^ **(n = 16)**	275 ± 39	6.9	0.93 [0.82–0.98]	0.91	10.3 [7.7–15.7]	0.19 [0.00–0.38]	5.5 [4.0–8.7]

**Table 4:** Mean difference (MD), effect size (ES), and p-value comparing the influence of graded exercise test stage length on all 14 lactate threshold methods.

		3 vs. 4	3 vs. 7	3 vs. 10	4 vs. 7	4 vs. 10	7 vs. 10
**Log-log LT**	**MD (W)**	10	12	15	2	6	3
** **	**ES**	0.24	0.28	0.36	0.05	0.14	0.08
** **	**p-value**	0.09	0.02	0.02	0.63	0.15	0.47
**OBLA 4.0 mmol**^.^**L**^**-1**^	**MD (W)**	15	24	27	9	12	3
** **	**ES**	0.34	0.56	0.63	0.22	0.29	0.06
** **	**p-value**	0.00	0.00	0.00	0.05	0.01	0.35
**OBLA 3.5 mmol**^.^**L**^**-1**^	**MD (W)**	15	23	25	8	11	3
** **	**ES**	0.34	0.53	0.60	0.20	0.26	0.06
** **	**p-value**	0.00	0.00	0.00	0.09	0.02	0.35
**OBLA 3.0 mmol**^.^**L**^**-1**^	**MD (W)**	14	21	24	7	9	3
** **	**ES**	0.34	0.50	0.57	0.16	0.23	0.06
** **	**p-value**	0.00	0.00	0.00	0.16	0.05	0.36
**OBLA 2.5 mmol**^.^**L**^**-1**^	**MD (W)**	14	19	21	5	7	2
** **	**ES**	0.34	0.46	0.53	0.12	0.18	0.06
** **	**p-value**	0.00	0.00	0.00	0.30	0.13	0.39
**OBLA 2.0 mmol**^.^**L**^**-1**^	**MD (W)**	13	15	18	2	4	2
** **	**ES**	0.33	0.38	0.45	0.06	0.12	0.06
** **	**p-value**	0.01	0.01	0.00	0.63	0.36	0.45
**Baseline + 0.5 mmol**^.^**L**^**-1**^	**MD (W)**	6	7	10	1	4	3
** **	**ES**	0.16	0.18	0.27	0.03	0.10	0.07
** **	**p-value**	0.25	0.27	0.10	0.85	0.46	0.50
**Baseline + 1.0 mmol**^.^**L**^**-1**^	**MD (W)**	16	18	20	3	4	1
** **	**ES**	0.40	0.47	0.51	0.07	0.10	0.03
** **	**p-value**	0.01	0.00	0.00	0.53	0.41	0.71
**Baseline + 1.5 mmol**^.^**L**^**-1**^	**MD (W)**	16	21	23	5	7	2
** **	**ES**	0.39	0.52	0.57	0.12	0.17	0.05
** **	**p-value**	0.00	0.00	0.00	0.27	0.14	0.49
**Dmax**	**MD (W)**	13	23	30	10	17	7
** **	**ES**	0.38	0.71	0.90	0.29	0.49	0.22
** **	**p-value**	0.00	0.00	0.00	0.00	0.00	0.00
**Modified D**_**max**_	**MD (W)**	10	23	29	13	19	6
** **	**ES**	0.27	0.59	0.79	0.32	0.50	0.16
** **	**p-value**	0.01	0.00	0.00	0.01	0.00	0.06
**Log-Poly-ModD**_**max**_	**MD (W)**	14	25	32	11	18	7
** **	**ES**	0.35	0.62	0.78	0.26	0.43	0.18
** **	**p-value**	0.00	0.00	0.00	0.00	0.00	0.02
**Exp-D**_**max**_	**MD (W)**	14	23	29	9	15	6
** **	**ES**	0.38	0.66	0.82	0.26	0.42	0.17
** **	**p-value**	0.00	0.00	0.00	0.00	0.00	0.02
**Log-Exp-ModD**_**max**_	**MD (W)**	15	26	33	11	18	7
	**ES**	0.35	0.64	0.80	0.28	0.44	0.17
	**p-value**	0.00	0.00	0.00	0.00	0.00	0.01

### ${\dot{W}}_{\max}$ and ${\dot{V}O}_{2\max}$

There was an inverse relationship between GXT duration and both ${\dot{W}}_{\max}$ and ${\dot{V}O}_{2\mathrm{peak}}$ (Table 5). The ${\dot{V}O}_{2\mathrm{peak}}$ values derived from GXT_3_ and GXT_4_ were similar to the ${\dot{V}O}_{2\mathrm{peak}}$ measured during GXT_1_ (Table 6); however, the values were outside the variability of the measurement (CV > 3%) [27]. ${\dot{V}O}_{2\mathrm{peak}}$ values from GXT_1_ and the corresponding VEB had the highest agreement (MD = 0.5 mL^.^kg^-1.^min^-1^, ICC = 0.96, SEM = 1.1 mL^.^kg^-1.^min^-1^ and CV = 2.0%) compared with any GXT and corresponding VEB. The remaining GXTs and corresponding VEB had a CV of 3.3, 2.0, 3.5 and 5.2%, for GXT_3_, GXT_4_, GXT_7_ and GXT_10_, respectively. The VEB performed following the longer duration GXTs (GXT_3-10_) underestimated the ${\dot{V}O}_{2\mathrm{peak}}$ from GXT_1_ (Table 6).

**Table 5:** Mean and standard deviation of ${\dot{V}O}_{2\max}$—highest measured ${\dot{V}O}_{2}$ during any graded exercise test (GXT); GXT ${\dot{V}O}_{2}$ -highest measured ${\dot{V}O}_{2}$ during each GXT; VEB ${\dot{V}O}_{2}$ highest measured ${\dot{V}O}_{2}$ during each verification exhaustive bout (VEB); ${\dot{V}O}_{2\mathrm{peak}}$, highest measured ${\dot{V}O}_{2}$ during either the GXT or corresponding VEB. Mean and standard deviation of GXT duration, max power (Watts) from each GXT, percentage of maximum power from the prolonged GXT expressed as a percentage of W maximum power from GXT_1_ and power of each VEB (Watts) from the GXTs. Relative power of the verification exhaustive bout expressed as a percentage (%) of the maximal power measured during the GXT. The subscript (i.e., 1, 3, 4, 7 or 10) refers to the stage duration (minutes) for each test.

	GXT_1_	GXT_3_	GXT_4_	GXT_7_	GXT_10_
${\dot{V}O}_{2\max}$ **(mL**^.^**kg**^**-1.**^**min**^**-1**^**)**			62.1 ± 5.8		
**GXT** ${\dot{V}O}_{2}$ **(mL**^.^**kg**^**-1.**^**min**^**-1**^**)**	60.6 ± 5.4	58.2 ± 5.3	57.3 ± 5.7	56.4 ± 5.2	54.9 ± 4.9
**VEB** ${\dot{V}O}_{2}$ **(mL**^.^**kg**^**-1.**^**min**^**-1**^**)**	60.1 ± 5.8	58.9 ± 5.9	58.8 ± 6.1	56.4 ± 5.9	54.7 ± 6.6
${\dot{V}O}_{2\mathrm{peak}}$ **(mL**^.^**kg**^**-1.**^**min**^**-1**^**)**	61.0 ± 5.3	59.7 ± 5.4	58.9 ± 6.0	57.3 ± 5.4	56.2 ± 5.5
**GXT Duration (min)**	11.3 ± 0.9	26.8 ± 1.4	34.9 ± 1.9	59.2 ± 3.3	81.6 ± 4.6
**Maximum Power (Watts)**	420 ± 55	337 ± 46	321 ± 47	303 ± 43	295 ± 43
**Percent** ${\dot{W}}_{\max}$ **of GXT**_**1**_ **(%)**	100	80.3 ± 2.9	76.4 ± 3.1	72.1 ± 3.6	70.3 ± 4.0
**VEB (Watts)**			378 ± 50		
**VEB (% of GXT** ${\dot{W}}_{\max}$**)**	90	109.7 ± 3.8	118.4 ± 18.7	125.4 ± 19.3	128.8 ± 20.4

**Table 6:** Mean difference (MD) and standard deviation, effect size (ES), coefficient of the variation (CV) and p-value (p) for the measured ${\dot{V}O}_{2\mathrm{peak}}$ values from GXT_1_ compared with the ${\dot{V}O}_{2\mathrm{peak}}$ values from GXT_3_, GXT_4_, GXT_7_, and GXT_10_ and for the ${\dot{V}O}_{2\mathrm{peak}}$ values from GXT_1_ compared with the ${\dot{V}O}_{2\mathrm{peak}}$ values from the VEB following GXT_3_, GXT_4_, GXT_7_, and GXT_10_. The subscript (i.e., 1, 3, 4, 7 or 10) refers to the stage duration (minutes) for each test.

	**GXT**_**1**_ **vs. GXT**_**3**_	**GXT**_**1**_ **vs. GXT**_**4**_	**GXT**_**1**_ **vs. GXT**_**7**_	**GXT**_**1**_ **vs. GXT**_**10**_
**MD (mL**^.^**kg**^**-1.**^**min**^**-1**^**)**	-1.2 ± 3.3	-2.1 ± 4.2	-3.7 ± 4.7	-4.8 ± 3.7
**ES**	0.23	0.36	0.69	0.88
**CV (%)**	3.8	4.9	5.6	4.6
**p**	0.13	0.06	< 0.01	< 0.01
	**GXT**_**1**_ **vs. VEB GXT**_**3**_	**GXT**_**1**_ **vs. VEB GXT**_**4**_	**GXT**_**1**_ **vs. VEB GXT**_**7**_	**GXT**_**1**_ **vs. VEB GXT**_**10**_
**MD (mL**^.^**kg**^**-1.**^**min**^**-1**^**)**	-2.1 ± 5.9	-2.1 ± 6.1	-4.6 ± 5.9	-6.2 ± 6.6
**ES**	0.37	0.37	0.81	1.04
**CV (%)**	4.2	4.9	6.1	5.9
**p**	0.02	0.98	0.03	0.03

## Discussion

The main findings of the present study are as follows. Only 11 of the 58 threshold values met our inclusion criteria as valid estimates of the MLSS. Of the 11 methods included in our analysis, three of the ModD_max_ methods yielded the most favourable estimations of the MLSS, and the Log-Poly-ModD_max_ derived from GXT_4_ provided the best estimation of the MLSS. There was an inverse relationship between stage length and LT, and this effect was larger in all D_max_ methods compared with the OBLA and baseline plus absolute lactate value methods. The ${\dot{V}O}_{2\mathrm{peak}}$ values measured during the longer duration GXTs (GXT_3-10_) underestimated the ${\dot{V}O}_{2\max}$ and the ${\dot{V}O}_{2\mathrm{peak}}$ values obtained from GXT_1_ (MD = 1.2 to 4.8 mL^.^kg^-1.^min^-1^). Finally, contrary to our hypothesis, the VEB after the longer duration GXTs did not yield ${\dot{V}O}_{2\mathrm{peak}}$ values comparable to the ${\dot{V}O}_{2\mathrm{peak}}$ derived from GXT_1_.

The use of five GXT protocols, 14 common LT methods, the RCP and RCP_MLSS_ resulted in 58 unique thresholds. However, despite their common use, we observed that only 11 of these values met our criteria for inclusion (MD < 7.9 W; ES < 0.2; r > 0.90). Of the four D_max_ methods included in our analysis, one consisted of the traditional ModD_max_ method [22]. This had the poorest agreement relative to the other ModD_max_ methods included in our analysis. The remaining three D_max_ methods are new variations of the ModD_max_ method, and the Log-Poly-ModD_max_ derived from GXT_4_ had the highest correlation and lowest mean difference with the MLSS. These variations of the ModDmax method use the power at the log-log LT as the initial intensity to calculate the ModD_max_ and then either the traditional third-order polynomial or exponential plus-constant regression curve to fit the lactate curve [23, 41]. Although the validity of these three methods has not previously been assessed, the favourable estimations of the MLSS may be related to the greater objectivity with which they determine the intensity that corresponds with the initial rise in blood lactate concentration [37].

Although the original D_max_ method is a commonly cited method for determining the LT [23], we observed large mean differences (19 to 49 W) between the D_max_ and MLSS. Three previous studies have purported to investigate the validity of this method to estimate the MLSS in trained male cyclists [15, 52, 53]. One concluded that the D_max_ method derived from GXT_3_ was a valid estimation of the MLSS (r = 0.97) [54]. We also observed a high correlation between D_max_ and the MLSS (r = 0.94 to 0.97) (Table 1), but, as indicated by the MD and other measures, a high correlation is not sufficient to establish validity [55]. Another study examined D_max_ derived from two GXTs with similar durations (36 vs. 39 min), but with different stage lengths (30-s vs. 6-min) [15]. The D_max_ derived from GXT_30s_ was not correlated (r = 0.51) with the MLSS, even though the MD was 5 W, whilst the D_max_ derived from GXT_6_ was correlated (r = 0.85); however, it underestimated the MLSS (MD = 22 W). The third study concluded the D_max_ derived from GXT_1_ yielded poor estimates of the MLSS (r = 0.56; bias = -1.8 ± 38.1 W) [53]. Thus, although some studies [15, 54] have used correlation analysis to suggest the D_max_ provides a valid estimate of the MLSS, this is not supported by the more comprehensive assessment of validity performed in the present and other studies [53].

There were five fixed blood LT methods and one baseline plus an absolute value that met our inclusion criteria, and, as previously reported [15, 24], these varied with the GXT protocol used. The baseline + 1.5 mmol^.^L^-1^ was the only LT derived from GXT_3_ included in our analysis (bias = -6 ± 35 W). This is consistent with the results of one previous study (bias = 0.5 ± 24 W), which also recruited trained male cyclists and had a similar GXT protocol design [56]. Consistent with our findings, this study also reported that an OBLA of 3.5 mmol^.^L^-1^ derived from GXT_3_ did not provide a valid estimation of the MLSS. In contrast, another study confirmed the validity of the OBLA of 3.5 mmol^.^L^-1^ [52], despite recruiting trained cyclists and using an identical GXT protocol. These conflicting results are likely attributable to the low reproducibility of the OBLA methods [16].

While none of the OBLAs from GXT_3_ met our inclusion criteria, the OBLA methods of 2.5 mmol^.^L^-1^ derived from GXT_4_ and GXT_7_ provided valid estimations of the MLSS, as did the OBLA of 3.0 mmol^.^L^-1^ derived from GXT_7_ and GXT_10_. The OBLA of 3.5 mmol^.^L^-1^ from GXT_10_ was the highest fixed blood LT that identified the MLSS. There is no previous data investigating the validity of these OBLA methods. However, it is worth noting that these five methods provided superior estimations of the MLSS compared with the original ModD_max_, but were less favourable than the newly-developed ModD_max_ methods.

An OBLA of 4.0 mmol^.^L^-1^ is the most commonly-accepted fixed blood lactate value for estimating the LT or MLSS. Three previous studies have attempted to validate use of an OBLA of 4.0 mmol^.^L^-1^ with cycle ergometry [15, 53, 57]. One study found that it overestimated the MLSS (MD = 49 W) when derived from GXT_1_ [53]. The other study reported poor agreement (bias 7 ± 49 W) when OBLA of 4.0 mmol^.^L^-1^ was derived from GXT_4_ [57]. The final study observed a poor correlation between an OBLA of 4.0 mmol^.^L^-1^ and the MLSS (r = 0.71) [15]. Our results indicated the OBLA of 4.0 mmol^.^L^-1^ overestimated the MLSS across all GXTs. Thus, in agreement with previous research, our results indicate; the OBLA of 4.0 mmol^.^L^-1^ does not accurately estimate the MLSS. It is also worth noting that the original authors cautioned the use of this OBLA method, given the lack of a significant correlation when comparing OBLA methods from a GXT and the MLSS [24].

The RCP derived from an 8- to 12-minute GXT consistently overestimates the MLSS [44, 53], and this was confirmed in our study (Table 1). Therefore, we used a regression equation based on the RCP (RCP_MLSS_) (Eq 5) to estimate the starting intensity for establishing the MLSS [33]. Our results indicate there was good agreement between the MLSS and RCP_MLSS_ (Table 3). Nonetheless, for many participants the difference between MLSS and RCP_MLSS_ exceeded the CV% for the MLSS (Fig 3). Therefore, although the RCP_MLSS_ can be used as a convenient ‘starting point’ when establishing the MLSS, we recommend methods based on blood sampling from the current study and assessing blood lactate kinetics in real time as recommended by Hering et al. [58] for a more accurate estimation of the MLSS.

Although a single GXT can be used to estimate both ${\dot{V}O}_{2\max}$ and LT, the optimal test duration for each measure is different [11, 13]. To address this challenge, we added a supramaximal VEB after each GXT, equivalent to that performed following GXT_1_, expecting all VEBs would yield similar ${\dot{V}O}_{2}$ values. However, the ${\dot{V}O}_{2\mathrm{peak}}$ values from the VEB after the longer duration GXTs underestimated the ${\dot{V}O}_{2\mathrm{peak}}$ from GXT_1_. Although the ${\dot{V}O}_{2\mathrm{peak}}$ values from GXT_3_ and GXT_4_ were similar to GXT_1_, the differences were larger than the typical coefficient of variability for ${\dot{V}O}_{2\mathrm{peak}}$ (CV < 3%) [59]. Our results are consistent with previous recommendations that longer duration GXTs are not optimal for establishing ${\dot{V}O}_{2\mathrm{peak}}$ [10, 60]. Furthermore, while a VEB can be used to verify that ${\dot{V}O}_{2\mathrm{peak}}$ was achieved, it appears that a VEB following a prolonged GXT cannot be used to establish ${\dot{V}O}_{2\max}$.

Extending the duration of the GXT stages results in a lower ${\dot{W}}_{\max}$ [61]. This has implications for exercise prescription, as it is common in sport and exercise science research to prescribe exercise intensity as a percentage of ${\dot{W}}_{\max}$. For example, in the present study the MLSS ranged from 63 ± 4% (range = 52 to 72%) of ${\dot{W}}_{\max}$ from GXT_1_ to 82 ± 4% (range = 74 to 88%) of ${\dot{W}}_{\max}$ from GXT_4_. Prescribing exercise in the current study cohort at a fixed percentage of ${\dot{W}}_{\max}$ (e.g., 73% of ${\dot{W}}_{\max}$), would result in all participants exercising above or below the MLSS, GXT_1_ and GXT_4_, respectively. This is important as it has previously been reported that prescribing exercise relative to LT results in a more homogenous physiological response than when exercise performed relative to ${\dot{W}}_{\max}$ [62]. This also highlights why it is important to consider the GXT protocol and the method used to determine relative exercise intensity when comparing results between studies.

The wide range of ${\dot{W}}_{\max}$ for each GXT is also note-worthy, the ${\dot{W}}_{\max}$ range for GXT_1_ was 320 to 517 W and the duration ranged from 9 to 12 minutes. Had we employed a standardized GXT (e.g., 35 W increments), and assuming ${\dot{W}}_{\max}$ stayed constant, the range would have been 9- to 15 min. Applying this to our longer duration GXTs resulted in a homogenous duration (GXT_4_: 32- to 39 min), whereas a standardised approach (e.g., 35 W increments) would have resulted in a range of 27- to 46 min [57]. Thus, individualizing GXT protocol design is a useful approach to ensure homogenous test duration [17].

## Conclusion

In conclusion, the traditional D_max_ and OBLA of 4.0 mmol^.^L^-1^ did not provide valid estimates of the MLSS. The best estimation of the MLSS was the Log-Poly-ModD_max_ derived from GXT_4_. The validity of our newly-developed ModD_max_ model may relate to the objectivity for determining the initial rise in blood lactate concentration. However, we must advise caution with the use of our newly-developed method until future research investigates the reliability and reproducibility. It is apparent that both ${\dot{V}O}_{2\max}$ and LT cannot be determined in a single GXT, even if the GXT is followed by a VEB. Therefore, to appropriately determine ${\dot{V}O}_{2\max}$ the optimum duration of a GXT is 8–12 minutes and the ${\dot{V}O}_{2}$ values measured during the GXT and VEB be within 3% = CV [63]. Our data also highlight how differences in GXT protocol design and methods used to calculate the relative exercise intensity may contribute to the conflicting findings reported in the literature.

## Abbreviations

LT: lactate threshold
GXT: graded exercise testing
${\dot{V}O}_{2\max}$: maximal oxygen uptake
OBLA: onset of fixed blood lactate accumulation
MLSS: maximal lactate steady state
VEB: verification exhaustive bout
BMI: body mass index
${\dot{W}}_{\max}$: maximum power output
${\dot{V}O}_{2\mathrm{pea}}$: highest measured oxygen uptake value
RCP: respiratory compensation point
RCP_MLSS_: estimate of the maximal lactate steady state via respiratory compensation point
B + mmol^.^L^-1^: blood lactate concentration increases above baseline value(s)
ModD_max_: modified D_max_
Exp-D_max_: exponential D_max_
Log-Poly-ModD_max_: Log-log Modified D_max_
Log-Exp-ModD_max_: Log-log Exponential Modified D_max_
CV: coefficient of the variation
ES: effect size
r: Pearson product moment correlation
ICC: intraclass correlation
SEM: standard error of the measurement
